# Bottlenecks in the transmission of porcine reproductive and respiratory syndrome virus (PRRSV1) to naïve pigs and the quasi-species variation of the virus during infection in vaccinated pigs

**DOI:** 10.1186/s13567-018-0603-1

**Published:** 2018-10-19

**Authors:** Martí Cortey, Gastón Arocena, Emanuela Pileri, Gerard Martín-Valls, Enric Mateu

**Affiliations:** 1 Departament de Sanitat i d’Anatomia Animals, Universitat Autònoma de Barcelona, 08193 Cerdanyola del Vallès, Spain; 2 IRTA, Centre de Recerca en Sanitat Animal (CReSA, IRTA-UAB), Campus de la Universitat Autònoma de Barcelona, 08193 Cerdanyola del Vallès, Spain

## Abstract

This paper describes the results of two experiments regarding porcine reproductive and respiratory syndrome virus (PRRSV1): the first one studied the existence of bottlenecks in an experimental one-to-one model of transmission in pigs; while the second analysed the differences between viral quasi-species in vaccinated pigs that developed shorter or longer viraemias after natural challenge. Serum samples, as well as the initial inoculum, were deep-sequenced and a viral quasi-species was constructed per sample. For the first experiment, the results consistently reported a reduction in the quasi-species diversity after a transmission event, pointing to the existence of bottlenecks during PRRSV1 transmission. However, despite the identified preferred and un-preferred transmitted variants not being randomly distributed along the virus genome, it was not possible to identify any variant producing a structural change in any viral protein. In contrast, the mutations identified in GP2, nsp9 and M of the second experiment pointed to changes in the amino acid charges and the viral RNA-dependent RNA polymerase structure. The fact that the affected proteins are known targets of the immunity against PRRSV, plus the differential level of neutralizing antibodies present in pigs developing short or long viraemias, suggests that the immune response selected those changes.

## Introduction

The comprehension of how the transmission of pathogens occurs is key to the understanding of infectious diseases. Most often, the source of excretion, the route of transmission, the portal of entry and the minimum infective dose, when known, are common features to characterize transmission. However, it is increasingly evident that transmission is an extremely complex phenomenon; for example, the existence of bottlenecks during host-to-host transmission [1]. A bottleneck can be defined as a sharp reduction in size (population bottleneck) or diversity (genetic bottleneck) in a population. Focusing on pathogen transmission, the existence of such bottlenecks must be examined with consideration to the portal of entry in the recipient host and the pathogen source (blood, nasal secretion, faeces, etc.). Since a pathogen may be present in different tissues, organs, or fluids, each one might be considered a compartment with its own particularities. The pathogen population contained in the compartments where the transmission to the next host occurs is termed transmissible population; whereas the successful infectors in the recipient host are called founder variants or transmission founders. The location, size, and genetic diversity of the transmissible population can influence the founder population after a transmission event [2–4].

Successful transmission founders can be thought of as either the result of a non-selective bottleneck—the particles that crossed by chance the portal of entry—, or viewed as a selective bottleneck, where only the variants fit enough to cross the portal of entry are transmitted. In the case of RNA viruses, which exist as quasi-species, these different scenarios could imply very different outcomes. On the one hand, a non-directional unspecific bottleneck would produce a new quasi-species cloud from randomly selected variants. In contrast, on the other hand, a directional bottleneck would promote the expansion in the recipient host of variants derived from founders fit for transmission that are subsequently selected, since they are not necessarily the fittest, neither the most efficient for replication in the host.

There are other factors, such as the immune status of the host that may influence the diversity of a quasi-species for example, in *Human Immunodeficiency Virus* (HIV) [5], *Influenza A Virus* [6] and *Hepatitis C virus* (HCV) [7], continuous diversification has been considered the means by which the virus escapes the immune system and establishes a persistent chronic infection. However, other additional factors, such as the antigenic cooperation between intra-host variants, may permit immune adaptation, leading to the co-existence of viral variants with different capacities to bind antibodies or to be attacked by the cell-mediated immunity [8, 9].

The ex vivo study of founder variants and the quasi-species evolution in humans is challenged by the difficulty of determining the precise timing of transmission and the associated quasi-species distribution in the donor. However, in animal models this can be examined in a more controlled environment. As a result, transmission bottlenecks and quasi-species variation can be more precisely determined.

Porcine reproductive and respiratory syndrome (PRRS) is one of the most economically detrimental pig diseases. It is caused by PRRS virus (PRRSV), a positive-sense, single-stranded RNA virus in the *Arteriviridae* family within the order *Nidovirales*, which exhibits one of the highest substitution rates observed [10, 11]. Experimental models to study PRRSV are well known and have been used in transmission studies [12, 13]. The immune response against PRRSV is unusual, since neutralizing antibodies appear late and cell-mediated immunity has an erratic course lasting weeks. However, after several weeks of viraemia, the virus is confined to the lymphoid tissue and eventually cleared [14, 15]. Neutralizing antibodies may protect against the homologous infection in a dose dependent way [16], although heterologous protection cannot be predicted [17]. In addition, there is a large individual variation in the immune response [18]. As a result, when a vaccinated animal is challenged with a heterologous strain, viraemia usually develops, but generally of shorter duration than in a naïve animal.

In the present study we used Next Generation Sequencing (NGS) to analyse the quasi-species diversity and evolution in a transmission model of PRRSV in order to: (i) characterise and compare the transmissible population and the founder variants in intra-nasally inoculated and naturally infected animals, (ii) compare the diversity at early and late phases of viraemia and, (iii) identify the differences in the viral quasi-species between vaccinated pigs developing short and long viraemias after being in contact with infected pigs.

## Materials and methods

### Animal experiment

**Figure 1:**
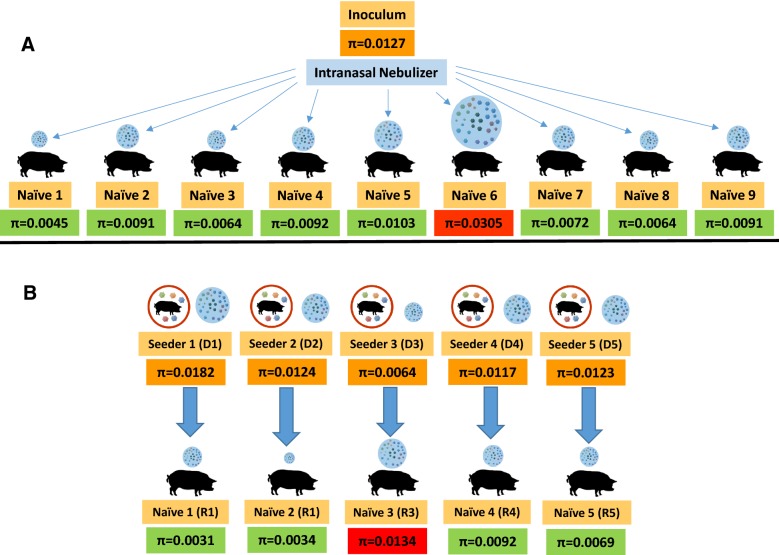
**Summary of the 14 transmission events studied.** **A** Intranasal inoculation of 9 naïve pigs using a nebulizer; **B** Experimental infection in a 1:1 basis of 5 non-vaccinated naïve pigs (R1 to R5) from seeders (D1 to D5). Nucleotide diversity (π) estimations of the donor population (orange boxes) and the founder variants, in green boxes if the global diversity decreased and in red if an increase was reported. The donor animals, the recipient animals, and the nucleotide diversity estimations are also depicted in Table 1.

Samples used in the present study were obtained in the course of a previous experiment aimed to determine the transmission of PRSRV in a one-to-one basis [12]. Table 1 and Figure 1 summarizes the first experiment regarding transmission, where two different scenarios were examined. The first one studied animals experimentally infected by the intranasal route with a PRRSV inoculum produced in Porcine Alveolar Macrophages (PAM) (*n* = 9); the second scenario studied cases of transmission by contact in naïve pigs (*n* = 5). In all cases, the serum samples used were collected on the first observed day of viraemia in the recipient, usually day 2 after inoculation or contact. When transmission occurred by contact, the donors’ serum of a sampling point prior to the transmission event (usually 1–3 days before the onset of the viraemia in the recipient) was analysed. Additionally, since samples at later viraemia stages were available, we examined the changes in the diversity throughout the viraemic period. Oral fluid samples were also available and might have been more representative than sera to characterize the transmissible quasi-species population. However, the viral load present in those samples was insufficient—even after cell passage—to carry out a successful NGS analysis.

**Table 1:** Variation of the viral load and nucleotide diversity (π) in transmission events and between days of viremia

Group	Animal n°	Viremic day	Viral load	π	Difference between donor and recipient	Difference between consecutive samples
Inoculum produced in PAM	N.A.			0.0127		
Inoculated animals	D1	1	7.91E+06	0.0045	−0.0082	
		*6*	*6.04E*+*05*	*0.0182*		+0.0137
		13	6.18E+06	0.0239		+0.0057
	D2	1	1.24E+05	0.0091	−0.0036	
		*6*	*3.23E*+*05*	*0.0124*		+0.0033
		13	1.24E+05	0.0115		−0.0009
	D3	*1*	*1.07E*+*06*	*0.0064*	−0.0063	
		13	1.15E+07	0.0075		+0.0011
	D4	*6*	*3.46E*+*05*	*0.0117*	−0.0010	
		13	2.14E+06	0.0528		+0.0411
	D5	*1*	*1.11E*+*06*	*0.0123*	−0.0004	
		13	6.09E+05	0.0132		+0.0009
	6	1	5.66E+05	0.0305	+0.0178	
		6	1.40E+07	0.0308		+0.0003
		15	1.97E+05	0.0097		−0.0211
	7	1	3.60E+06	0.0072	−0.0055	
		13	5.88E+05	0.0524		+0.0452
	8	1	5.71E+05	0.0064	−0.0063	
		06	7.37E+04	0.0152		+0.0088
		13	2.66E+06	0.0088		−0.0064
	9	1	4.81E+06	0.0091	−0.0036	
		6	2.58E+05	0.0041		−0.0050
		13	1.64E++06	0.0335		+0.0294
Naïve infected by contact	R1	1	1.23E+07	0.0031	−0.0151	
		6	4.60E+04	0.0119		+0.0088
		22	5.99E+03	0.0178		+0.0059
	R2	1	1.63E+06	0.0108	−0.0090	
		8	2.41E+07	0.0091		−0.0017
		15	2.25E+03	0.0109		+0.0018
	R3	3	2.76E+05	0.0134	+0.007	
		10	8.57E+06	0.0165		−0.0031
	R4	1	3.17E+06	0.0092	−0.0025	
		5	2.90E+08	0.0065		+0.0027
	R5	3	6.06E+06	0.0069	−0.0054	
		10	5.97E+06	0.0099		+0.0030

The table shows the difference in nucleotide diversity between: (i) the transmissible population in sera for the donor before the transmission and the founders in the recipient at the first day of viremia, and (ii) samples of the same animals in different days of the virological course. The difference of nucleotide diversities was calculated by subtracting the π value of a given day from the π value the previous examined day. For the first day of each animal the difference was calculated with regards to the inoculum (inoculated animals) or with regards to the diversity of the donor (D1 to D5) on the likely day of transmission (transmission by contact) to the corresponding recipient (R1 to R5) animals. In italic, the most likely day of transmission for the donor animals.

In a second experiment, the changes in the viral quasi-species present in blood of vaccinated animals that were infected by contact with seeder pigs were examined (*n* = 11). For this purpose, the last day of viraemia was analysed. In this later experiment, animals were seen to develop long viraemias (≥ 7 days) or short viraemias (< 7 days) and were classified accordingly into two groups: short viraemia (*SV*, *n* = 7) or long viraemia (*LV*, *n* = 4). Since in some cases the amount of virus in blood was not enough to proceed directly to NGS, all samples were subjected to a single passage in PAMs in order to maintain similar conditions for all.

For the first experiment, the inoculum used was a sixth passage of strain CReSA3267 (Accession Number JF276435). The percentage of nucleotide differences between the quasi-species present in the inoculum and the one in the vaccine batch was 10.6%. In both experiments, viral load in blood was determined by one step RT-PCR (qRT-PCR) targeting PRRSV ORF7 using the method described by Pileri et al. [19].

### NGS protocol

As stated above, all sera were previously passaged once in PAM. Cell culture supernatants were firstly centrifuged up to 14 000* g* in order to remove potential debris. Afterwards, total RNA was extracted using the Trizol LS© reagent following the manufacturer’s instructions. Extracted RNA was assessed by spectrophotometry at 260 nm and 280 nm and used in the PRRSV-specific qRT-PCR as previously stated for determining concentration of RNA and purity.

The assessment of PRRSV diversity within each sample was characterised directly from RNA without any previous amplification step using a NGS approach developed by our group. The procedures included: (i) construction of a genomic library for Illumina NGS sequencing using a commercial protocol and reagents (Protocol for use with Purified mRNA or rRNA Depleted RNA and NEBNext^®^ Ultra™ II RNA Library Prep Kit for Illumina^®^, New England Biolabs), (ii) trimming of low quality reads (QC > 20 as determined by FastQC©software, Babraham informatics) using Trimmomatic© [20], (iii) mapping of the reads against strain CReSA3267 using the Burrows-Wheeler Aligner applying the BWA-MEM algorithm for long reads [21], (iv) variant calling with SnpSift© to determine the frequency of each nucleotide at each position of the reference genome and, (v) construction of the viral quasi-species in fasta format.

### Validation of the procedure, estimation of the PAM passage error rate and quality control check

Given that the samples used were cell culture supernatants, it was necessary to validate the technique for ascertaining the potential bias introduced between PAM-passaged and un-passaged samples. For this purpose, four serum samples with high viral load (> 10^6^ viral genomes/mL after quantification by qRT-PCR) were directly deep-sequenced. In parallel, the same four sera were single passaged in PAM during 72–96 h and the cell culture supernatants were also deep-sequenced. Both samples were then filtered against the reference genome and a quasi-species for each one was constructed. Finally, both quasi-species were compared. To be admitted to further analysis, a sample should produce a complete genome with at least a depth of 100 reads per nucleotide position.

### Nucleotide diversity estimations and frequency changes per position in the transmission events

To assess the change in the diversity of viral populations in the different transmission cases, the calculation of the nucleotide diversity (π) was performed using DNAsp [22]. These calculations were done comparing respectively: (a) the diversity in the PAM inoculum versus the diversity in the samples collected the first day of viraemia in experimentally inoculated animals and, (b) the diversity on the first day of viraemia of animals infected by contact versus the diversity in the sample of the donor on the most likely day of transmission. Additionally, with the transmission by contact cases (5 animals) the frequency of each nucleotide in each position in the donor and the recipient were compared to estimate what nucleotides increased or decreased their frequency in the transmission event. Frequency changes above 4.9% were arbitrarily considered relevant.

### Analysis of molecular variance (AMOVA) in vaccinated animals

In the case of vaccinated animals, it was considered key to identify nucleotide positions that could be differentially selected in animals with longer or shorter viraemias. Therefore, firstly animals were grouped according to the viraemia as stated before (Long viraemias, LV, ≥ 7 days, or short viraemias, SV, < 7 days). Then, the average frequencies of each nucleotide per position and group were compared using AMOVA in Arlequin ver 3.5.2.2 [23]. Positions presenting a Fct > 0.05 were selected for further analysis.

### Screening of potential changes in the amino acid composition in the viral proteins

For all cases (transmission to naïve or vaccinated animals), the resulting reads were ordered to represent the different viral proteins known. Once this was done, the potential changes in codons for the positions showing changes above the considered threshold (4.9%) for frequencies of nucleotides were analysed.

As a first step, each change potentially causing the appearance or increase of a non-synonymous codon was annotated in the corresponding domain of the protein if known. Then, by bibliographic review, the affected positions were assessed for a known function. In addition, the changes in the protein structure were evaluated using SWISS-MODEL [24]. The 3D structures were built up choosing the default parameters in the program and the potential modification in the charge of the protein or site were evaluated.

## Results

### The RNA NGS method was suitable for assessing viral quasi-species

Deep sequencing results for PRRSV1, obtained from four un-passaged sera with high viral loads and from four cell culture supernatants of single-passaged samples from the same sera, produced similar viral quasi-species. The observed differences between the quasi-species, obtained from un-passaged sera and the isolated virus, ranged between 1 and 3 nucleotides for every 10 000 nucleotides. So, the results obtained from un-passaged sera or single-passaged isolates only differed at this error rate, which was considered an acceptable bias.

The quality scores (QC) of the NGS runs were above 30 in all the analysed samples (equivalent to equal or less than 0.1% error in the reads obtained), yielding a depth of reads for viral sequences above 115 in all cases. With this depth, variations in the range of percentage units could be determined.

### The characterization of PRRSV transmission events supports the existence of bottlenecks

The transmission experiment scheme and results are summarized in Table 1 and Figure 1. In 8/9 of the intra-nasally inoculated naïve pigs, the viral population in blood at the onset of viraemia showed lower diversity compared to the initial inoculum. Similarly, in 4/5 of the naïve pigs infected by direct contact with a seeder, the observed nucleotide diversity was lower compared with their seeder counterparts. As a whole, and assuming the limitations of the method, the results pointed to a reduction in the diversity of the founders compared to the transmissible population, supporting the existence of a bottleneck during PRRSV transmission events.

Taking advantage of the availability of serial samples during the virological course of each animal, it was possible to compare the diversity at different stages (Table 1). After the initial reduction during transmission, nucleotide diversity increased in most pigs analysed, although in some animals this was not the observed pattern.

### Preferred and un-preferred transmitted variants are differentially distributed along the PRRSV genome

**Figure 2:**
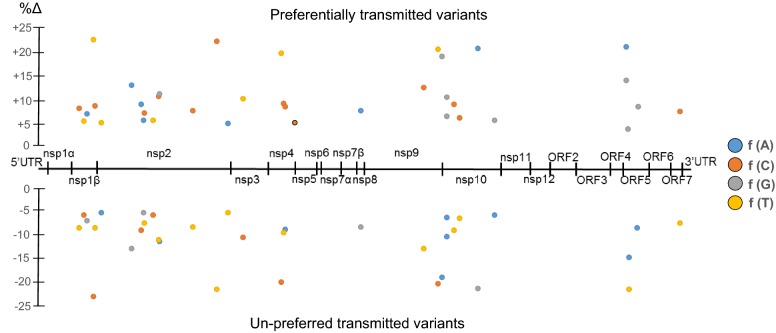
**Preferentially and un-preferred transmitted variants.** Mean percentage of variation in nucleotide frequencies along the PRRSV1 genome of the identified preferred or un-preferred transmitted variants in the transmission events between infected and naïve pigs studied (Figure 1B). Only positions with variations larger than 5% are shown.

Mean frequency differences for each nucleotide in every genome position were calculated for the transmission events where naïve acceptors were infected by seeder pigs (Figure 1B). For this section we used only data from pigs infected by contact, since for the inoculated pigs (Figure 1A), the PAM propagated inoculum might have had shown some level of adaptation to the cell passages and this might have masked the true changes associated with transmission fitness. Figure 2 shows the mean increase or decrease in the frequencies of each nucleotide per position between the quasi-species of the transmissible population and the transmitted founders (only values larger than 5% are shown). An increase in the nucleotide frequency was associated with a preferentially transmitted variant, while a decrease was identified as an un-preferred transmitted one. Along the PRRSV1 genome, 65 variants in 32 positions were detected (32 preferred and 33 un-preferred). Nineteen nucleotide variants produced synonymous changes, while 13 variants induced potential amino acid changes. These mutations were consistently reported in the quasi-species within both transmissible populations and transmitted founders, indicating that the mean differences reported were not caused by results observed in single transmission events.

A salient feature exhibited was the non-random distribution of the affected positions: 10 in nsp2 region (31%, 4 synonymous and 6 non-synonymous), 3 in nsp4 (9%, 2 synonymous and 1 non-synonymous), 3 in nsp9 (9%, all synonymous), 6 in nsp10 (19%, 3 synonymous and 3 non-synonymous) and 3 in ORF5 (9%, 1 synonymous and 2 non-synonymous).

Next, we examined and related the non-synonymous changes with the regions and known features of the encoded proteins. The six amino acid changes detected within nsp2 fell in the two hypervariable regions flanking the papain-like cysteine protease domain, with one change (Gln-336-Lys) located in the B Cell epitope site 3 proposed by Oleksiewicz et al. [25] and a second one (Tyr-736-His) falling in a non-conserved epitope inducing IFN-γ and IL-10 responses, as reported by Burgara-Estrella et al. [26]. In nsp4, an Ile-142-Leu change was located in the middle β-barrel domain II of the main PRRSV proteinase 3C-like protease, but the mutation did not result in any substantial change of the 3D structure of the protein. The single change detected in nsp5—Leu-32-Phe—was located in a transmembrane domain. Regarding nsp10, 2/3 amino acid changes identified in this protein fell in the Zinc-binding domain, while the third was located in the C-terminal domain. One of the affected residues, located at position 46, preferentially changed from Ser to Gly during the transmission events. Finally, in GP5 two nucleotide mutations in the first and second position of a codon led to the change Ile-36-Asp in the hypervariable ectodomain of GP5, just after a signal peptide cleavage site. None of the above-mentioned changes produced a relevant structural change in any protein.

### Differences in viral quasi-species between short- vs long-viraemic pigs are mostly located in nsp9 and ORF2

**Figure 3:**
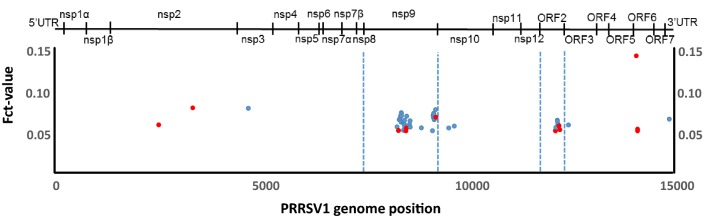
**Quasi-species differences between vaccinated animals.** Location of the nucleotide positions along the PRRSV1 genome showing differences between two groups of vaccinated animals developing short (*n* = 7) or long viraemia (*n* = 4) against PRRSV1 infection (calculated as Fct results, only values > 0.05 are shown). Synonymous changes are marked with blue dots, while red dots identify non-synonymous changes.

In PRRS, immunity against heterologous viral strains is considered partial and, consequently, the infection of vaccinated animals is possible if a heterologous strain is used. After examining transmission to naïve pigs, we compared the viral quasi-species in two groups of vaccinated pigs infected by contact through seeder penmates in a one-to-one basis. Those vaccinated animals developed viraemias that were classified as long (*LV* > 7 days) or short (*SV* < 7 days). The results indicated a different distribution of the changes in nucleotide frequencies between groups along the viral genome (Figure 3). As in the first experiment, mutations were consistently reported within *LV* and *SV* groups of quasi-species. Forty-five of the 55 positions (81.8%) identified were located in the nsp9 (37 positions, 64%) and ORF2 (8 positions, 15%); while these two proteins only account for 17.8% of the nucleotide positions in the viral genome. Remarkably, in all these 55 positions, the nucleotide variants characterising the *LV* group coincided with the nucleotide present in the original strain causing the infection, while the *SV* group always showed a different nucleotide. When the nucleotide variants present in the *SV* and *LV* were translated, most variants (43) generated the same amino acid (blue dots in Figure 3), but 12 introduced amino acid changes: 2 located in nsp2, 5 in nsp9, 3 in ORF2 and 2 in ORF6 (red dots in Figure 3).

In addition to this, both *SV* and *LV* groups shared an increase in the frequency of Lys at amino acid 106 of GP5 (13% in *SV* and 21% in *LV*), instead of the 106-Gly present at 100% in the initial inoculum.

**Figure 4:**
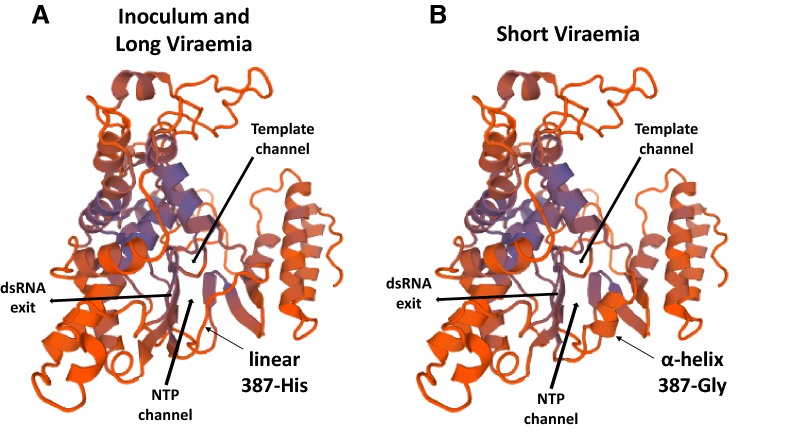
**Structural differences between RNA-dependent RNA polymerases.** 3D-structures of the PRRSV1 RdRp based on the translated sequences of nsp9 from the viral quasi-species of the inoculum (**A**), long (**A**) and short (**B**) viraemic pigs. The NTP and template channels, the dsRNA exit, and the amino acid residue 387 are indicated.

Again, non-synonymous changes were examined for potential biological significance. The two changes identified in nsp2, Ala-392-Thr and Pro-669-Ser, fell in the B-cell epitopes 4 and 7 described by Oleksiewicz et al. [25]. In nsp9, one of the key enzymes for PRRSV synthesis, all the amino acid changes identified were located in the viral RNA-dependent RNA polymerase (RdRp), encoded by the C-terminal domain. The comparison of the RdRp 3D structures between *SV* and *LV* groups (inferred with SWISS-MODEL) indicated that the amino acid changes Lys-338-Arg and Lys-641-Arg did not produce any evident structural change. On the contrary, the change Gly-387-His, caused by changes in the three nucleotide positions of the codon, induced a structural modification. The presence of a 387-Gly in *SV* group resulted in the formation of an α-helix between residues 384–388 and reduced the positive charge at the opposite side of the active centre of the protein, where Mg^2+^ molecules bind (Figure 4).

In GP2, the three amino acid changes identified, namely Gln-146-Arg, Val-151-Ala and His-184-Arg, fell in the ectodomain, slightly downstream the linear B-cell epitopes proposed by De Lima et al. [27] and Oleksiewicz et al. [28]. The amino acid changes reported for this protein implied more positively charged residues in *LV* compared to *SV* group.

Finally, the last two amino acid changes, Asp-10-Asn and Stop-25-Tyr, were located in the short stretch exposed at the virion surface in the N-terminal ectodomain of protein M, encoded by ORF6. Those positions, especially the Asp-10-Asn, were close to the residue 8-Cys that forms a disulphide bond with the 50-Cys of GP5 protein. The 10-Asp-Asn implies a charge modification from neutral (*LV*) to negative (*SV*).

## Discussion

With the introduction of NGS technologies, the experimental analysis of viral genetic diversity has changed dramatically. Due to its massively parallel approach, NGS generates millions of reads that cover every nucleotide position. Hence, low-frequency variants within viral quasi-species can be adequately detected and characterized [29] and it is possible to see variations that, although significant, would remain unnoticed using Sanger sequencing. However, the usefulness of NGS for viral diversity estimations depends crucially on the quality of the sample and on the procedure to prepare it [30]. In the present work we applied a tailor-made NGS method to characterize the diversity of PRRSV quasi-species without the need of primers. The method omitted the PCR amplification step and used instead a single passage in PAM when needed because of the low amount of virus present in many of the samples. By using PAM, the same cells that support viral replication in the host, a low bias was generated in a single passage, and the error rate produced was also very low. This approach could be useful for other viruses that cannot be analysed directly by NGS from biological samples because of the low titres present.

As stated, virus populations may face bottlenecks during the infection cycle [1]. When transmission takes place through a mucosal portal of entry, infection is usually initiated by a limited number of viral particles compared to the total number of particles reaching that portal [4]. Besides population bottlenecks, there are many examples of extreme genetic bottlenecks in RNA viruses such as HIV (see reviews by [4, 31], HCV [32–34] and *Simian immunodeficiency virus* [35].

In most of the experimentally inoculated pigs and the animals infected in a quasi-natural way by contact with infected seeder pigs, viral diversity was reduced during transmission, supporting the existence of a bottleneck during PRRSV infection. The nature of such a bottleneck is more difficult to determine. The changes in the nucleotide diversity were not scattered randomly across the viral genome but focused in a few targets. Beforehand, one could think that the structural proteins interacting with the target cells would be the most affected ones, since they are the first interacting with the mucosa surface. Interestingly, this was the case with GP5, the viral glycoprotein establishing the first interaction with porcine sialoadhesin, one of the viral receptors on the macrophage surface [36]. The preferentially transmitted variant introduced a change in position 36 favouring an Asp, a more acidic amino acid. It is difficult to interpret this result, but it is located close to a potential glycosylation site and adjacent to the neutralization epitope in GP5 [37]. It is tempting to hypothesize a potential increased interaction between GP5 and sialoadhesin favoured by the higher polarity of the 36-Asp variant. It is also worth noting that all but one of the other favoured changes affected non-structural proteins, pointing towards the selection of variants with different replication characteristics, although the result of the precise amino acid changes could not be ascertained from the literature.

After the initial diversity reduction during the transmission event, the viral diversity of the circulating quasi-species increased in most cases, as expected in an initial expansion of a viral population in a naïve animal. Afterwards, in later stages, diversity could increase or fall but since a detailed characterisation of the immune response at each timepoint was not performed, the causes are not clear.

The third objective of this work was to analyse the quasi-species differences between two groups of vaccinated pigs developing short and long viraemia. In the present work, the duration of the viraemia was correlated with the titres of neutralizing antibodies against the virus; *LV* pig titres ranged between 2 and 3 log2, while the observed values for *SV* pigs moved between 4 and 6 log2 [10]. Therefore, beforehand, main changes were expected to be located in potential targets for the neutralizing antibodies. Neutralizing antibodies for PRRSV have been reported to be induced by GP2, GP3, GP4, GP5 and M proteins [16, 38]. In the present case most of the changes in *SV* occurred in nsp9, followed by ORF2 that encodes GP2 (Figure 3). The 3D modelling of nsp9 (RdRp, Figure 4) showed that the introduction of a 387-Gly-His after mutations in the three nucleotides of the codon, produced a change in the folding of the protein, from linear to α-helix. This change resulted in the absence of a positively charged group opposite to the site of union of Mg^2+^, and would probably cause a modification in the electrostatic forces involved in the interaction with the NTP channel. It can be hypothesized that such a change would affect the efficiency of RNA synthesis; thus resulting in lower expression of the viral epitopes as a mechanism of escape. Unfortunately, with the analyses performed, this hypothesis cannot be proven.

Regarding the changes in the ORF2, the variants found in *SV* also resulted in a less charged protein. GP2 is known to build a complex with GP3 and GP4 that interacts with CD163 [39], the essential cell receptor for PRRSV [40, 41]. Again, it cannot be established if the changes in GP2 polarity reported modify any of these interactions, either with GP4 or CD163.

Apart from the aforementioned changes in nsp9 and ORF2, an additional interesting change was observed in the M protein encoded by ORF6. In the present study, for *SV* pigs, the mutation 10-Asp introduced a negative charge close to the residue 8-Cys that establishes a disulphide bond with the residue 50-Cys of GP5 [42]. GP5 and M form a heterodimer that interacts with heparan sulphate during PRRSV attachment; and later on, with sialoadhesin during the internalization of the virus [36, 43, 44]. The disruption of this bond correlated with the loss of viral infectivity in other members of the *Arteriviridae* family, such as *Lactate dehydrogenase*-*elevating virus* [45] and *Equine arteritis virus* [46]. The negative charge present in 10-Asp (*SV*) may interfere in the disulphide bond between GP5 and M. Therefore, potential weaker interactions with heparan-sulphate and sialoadhesin may be induced in the variants present in *SV* pigs. Another study showed that a single deletion adjacent to this disulphide bond produces the escape from a neutralizing antibody targeting an epitope located in GP5 [47]. It is tempting to think that this may be a similar case.

Interestingly, in both groups of vaccinated animals, compared to the initial inoculum a significant change favouring 106-Lys instead of 106-Gly was observed. The position 106 in PRRSV1 corresponds to 104 in PRRSV2. Fan et al. [48] showed that a substitution of 104-Gly by 104-Arg resulted in a decreased susceptibility to neutralization. Both Arg and Lys are hydrophilic amino acids of alkaline pK positively charged, while Gly is a non-charged amino acid. Therefore, it is reasonable to think that the change 106-Lys in PRRSV1 may act similarly to the 104-Arg in PRRSV2 and results in an escape mechanism in the presence of neutralizing antibodies.

Most of the mutations reported between *LV* and *SV* groups (10 out of 12) are commonly present among the PRRSV1 complete genomes available in GenBank, except the mutations 387-Gly in nsp9 and 25-Stop in ORF6—characteristic of the *SV* group—that are rarely reported.

In the context of animals with higher titres of neutralizing antibodies, as observed in the *SV* group [12], the changes reported in GP2 and M could be understood as escape mutations. The lower antibody titres in the *LV* group probably prevented those changes from being positively selected, and the major variants present in the initial inoculum remained the commonest. In contrast, changes in 106 of GP5 seem to be common to all analysed animals. It was proposed that T-cell responses contributed to partial levels of cross-protection in PRRSV, and therefore, the changes observed in nsp9 could be seen as escape variants [49], or as a means of escaping by producing very low levels of antigen. Potential T-cell epitopes for PRRSV have been proposed for nsp9 [50], as well as for the RdRp of other *Nidovirales* [51, 52]. It could be conjectured that those changes in nsp9, GP2 and M may result in less efficient viral variants for, either interaction with the cell receptor, or replication. This scenario would imply that in animals with higher levels of immunity, the variants escaping the immune system would not be fit enough to maintain a viable quasi-species cloud, and consequently, the viral population would collapse. Accordingly, nsp9, GP2 and M would be clear targets for new vaccine development. These results highlight the importance of the immune system of the host, and specifically the neutralizing antibodies, to efficiently combat and clear PRRSV infection. The role of neutralizing antibodies as a correlate of protective immunity against PRRSV is well known (reviewed in [16]).

In summary, the present report shows a feasible approach to study transmission events and changes in viral quasi-species during the course of an infection in pigs. The results were compatible with the existence of a transmission bottleneck for PRRSV and showed some targets for understanding the effects of the immune response on viral diversity. This pig model could be used to study human diseases such as influenza and to gain understanding of how transmission of RNA virus occurs.
